# Predictors of 2-Year Trajectory of Post-Traumatic Stress Disorder Following Physical Injury

**DOI:** 10.1155/2024/5570405

**Published:** 2024-09-02

**Authors:** Jae-Min Kim, Ju-Wan Kim, Hee-Ju Kang, Ye-Jin Kim, Hyunseok Jang, Jung-Chul Kim, Sung-Wan Kim, Il-Seon Shin, Robert Stewart

**Affiliations:** ^1^Department of Psychiatry, Chonnam National University Medical School, Gwangju, Republic of Korea; ^2^Division of Trauma, Department of Surgery, Chonnam National University Medical School and Hospital, Gwangju, Republic of Korea; ^3^King's College London (Institute of Psychiatry, Psychology and Neuroscience), London, UK; ^4^South London and Maudsley NHS Foundation Trust, London, UK

## Abstract

**Objectives:**

This study investigated post-traumatic stress disorder (PTSD) trajectories and their predictors over a 2-year period, in individuals recovering from physical injuries.

**Materials and Methods:**

Between June 2015 and January 2021, 1,142 patients from a South Korean University Hospital Trauma Center underwent baseline evaluations, including PTSD-related measures and sociodemographic characteristics. They were subsequently followed up for PTSD using the Clinician-Administered PTSD Scale at 3, 6, 12, and 24 months. The analyzed sample consisted of 1,014 patients who were followed up at least once after the baseline and 3-month evaluations. Latent class growth analysis and logistic regression models were used.

**Results:**

Five distinctive trajectories were identified: resilient, worsening/recovery, worsening, recovery, and chronic groups. The worsening/recovery trajectory was associated with previous traumatic events and traffic-related injuries, while the worsening trajectory was linked to higher education and elevated depressive symptoms. The recovery trajectory was characterized by female sex, childhood abuse, traffic-related injuries, dissociative subtype, and higher levels of anxiety and depressive symptoms. The chronic trajectory was predicted by the dissociative subtype and heightened anxiety symptoms.

**Conclusion:**

These findings highlighted the heterogeneity of PTSD symptom development and, thus, the importance of considering individual characteristics when assessing and addressing PTSD following physical injuries.

## 1. Introduction

Post-traumatic stress disorder (PTSD) is a multifaceted and incapacitating mental condition that often emerges in the aftermath of significant traumatic experiences. Among these, traumatic physical injuries resulting from accidents, acts of violence, or other distressing incidents rank prominently as triggers for PTSD development. Such injuries are closely linked to enduring impairments in functioning and a diminished quality of life [1]. The identification of predictors that contribute to the onset and progression of PTSD is an essential step toward both the prevention and management of this debilitating disorder. Therefore, many potential risk factors have been investigated, including sociodemographic variables (such as age, gender, education, and socioeconomic status), pre-existing health conditions (prior mental or physical disorders, past trauma, childhood adversities, substance abuse, personality traits), trauma-related attributes (like injury severity and dissociation), and peri-trauma states (psychological distress and cognitive symptoms, as well as physiological indicators like heart rate) [2, 3, 4].

Despite extensive investigations, findings on PTSD predictors have been highly discrepant. A comprehensive systematic review comprising 44 studies focused on PTSD predictors in survivors of road traffic accidents highlighted inconsistencies between studies, with conflicting outcomes on nearly all investigated variables [5]. This heterogeneity in findings could potentially be attributed not only to the fact that many studies have assessed PTSD at a single specific time point following the traumatic incident, spanning from 1 month to 2 years postevent [6, 7], but also to variations in the assessment methods employed across different studies.

Understanding the development of PTSD necessitates integrating predictive factors within a theoretical framework that accounts for the variability in its trajectories. The diathesis-stress model posits that PTSD results from the interaction between pre-existing vulnerabilities and traumatic stress, explaining the variability in PTSD trajectories—from resilience to chronic dysfunction—based on individual diatheses. Additionally, the conservation of resources theory suggests that trauma leads to PTSD by depleting critical coping resources, influencing trajectories of worsening or recovery. Biopsychosocial models further propose that the interplay of biological, psychological, and social factors determines the onset and course of PTSD, thereby influencing the trajectory an individual might follow [8].

Recent advancements in machine learning and quantitative forecasting have begun to offer new insights into varied trajectories of PTSD, such as symptom fluctuations, delayed onsets, and diverse recovery patterns. For instance, studies by Galatzer-Levy et al. [9, 10] and Tomas et al. [11] have applied machine learning techniques to forecast PTSD trajectories from early trauma responses, demonstrating how complex data-driven approaches can reveal comprehensive patterns of resilience and dysfunction following trauma. These patterns include distinct groups such as consistently low symptomatology (resilient), initial high symptoms followed by gradual improvement (recovery), and persistent high symptomatology (chronic). Similarly, Schultebraucks et al. [12, 13, 14] have contributed significant findings on predicting PTSD trajectories using a variety of biomedical and psychosocial data, which could potentially refine our understanding of PTSD development over time. Specially, Galatzer-Levy et al. [15] identified three distinct trajectories (nonremitting, slow remitting, and rapid remitting) during a 15-month follow-up, evaluating predictors from emergency room assessments and 10 days postevents between remitters and nonremitters. Furthermore, subsequent studies have identified four trajectories (resilient, delayed-onset, recovery, and nonremitting) over shorter follow-up periods of 6–12 months [11, 14]. One notable longitudinal study, spanning a span of 6 years, segmented the trajectories of PTSD into five distinct patterns—resilient, worsening, worsening/recovery, recovery, and chronic—and highlighted several baseline factors that appeared to predict poorer trajectories [16]. However, this investigation examined a limited array of predictive elements, focusing on sociodemographic factors and a handful of injury-related variables.

A comprehensive assessment of potential predictors, utilizing sociodemographic and clinical profiles primarily derived from self-reports, observer-rating scales, and electronic health records and encompassing both resilience and vulnerability at baseline, combined with an investigation of their longitudinal associations with different PTSD trajectories, could improve information on factors influencing the intricate courses of PTSD and have the potential to address existing gaps in knowledge. Using data obtained from a prospective 2-year study involving Korean patients with physical injuries, we aimed to investigate the associations between baseline factors and PTSD trajectories. We hypothesize that distinct PTSD trajectories will emerge and that these trajectories will be significantly associated with specific sociodemographic and clinical predictors.

## 2. Materials and Methods

### 2.1. Study Overview and Participants

This analysis constitutes an integral component of the biomarker-based diagnostic algorithm for post-traumatic syndrome (BioPTS) study, aimed at developing accurate models for the diagnosis and prediction of PTSD. Study details have been previously published in a protocol paper [17] and further discussed in relation to differential predictors of PTSD in a recent study [18]. In summary, the study's participants were prospectively enrolled from patients who had recently undergone hospitalization for physical injuries between June 2015 and January 2021 at the Department of Trauma Center of Chonnam National University Hospital (CNUH) in Gwangju, South Korea. The severity of injuries was assessed using the Injury Severity Score (ISS) [19] and the Glasgow Coma Scale (GCS) [20]. Study inclusion criteria comprised the following: (i) individuals aged 18 years or older at the index injury; (ii) patients hospitalized for more than 24 hr after sustaining a moderate to severe physical injury (ISS ≥ 9); and (iii) individuals sufficiently proficient in the Korean language to comprehend the study protocol. Exclusion criteria were as follows: (i) moderate or severe brain injury (GCS < 10); (ii) physical injuries resulting from suicide attempts; (iii) conditions hindering comprehensive psychiatric evaluation due to severe physical ailments; (iv) prior history of psychiatric disorders, including psychotic disorder, bipolar disorder, or alcohol or substance use disorders other than depressive and anxiety disorders; (v) significant cognitive impairments due to organic mental or neurocognitive disorders; and (vi) pre-existing convulsive disorders or a history of anticonvulsant use. Participants satisfying the eligibility criteria and expressing willingness to partake in the study underwent psychiatric assessments within 1 month of their hospitalization, conducted in person. Subsequent follow-up evaluations were conducted via telephone interviews at 3, 6, 12, and 24 months post the physical injury event. The culmination of patient visits occurred in January 2023. Ethical clearance for this study was obtained from the Chonnam National University Hospital Institutional Review Board (CNUH 2015-148). All participants meticulously reviewed the consent form, and written informed consent was duly acquired.

### 2.2. Baseline Evaluations

#### 2.2.1. Trauma and PTSD-Related Characteristics

To comprehensively assess trauma and PTSD-related characteristics at baseline, several key aspects were evaluated. The type of accidental injury was evaluated using the life events checklist (LEC) [21], which aided in identifying the specific type of traumatic event that participants had experienced. To streamline the analysis, given the number of participants, injury types were divided into four main categories: (i) traffic-related injuries, which include those sustained in automobile accidents, motorcycle crashes, and bicycle collisions; (ii) falls, referring to injuries from falling from heights such as ladders, stairs, or other elevated platforms; (iii) slips, encompassing injuries resulting from slipping sideways on wet or uneven surfaces; and (iv) other types of injuries, which cover a range of less common mechanisms not included in the aforementioned categories, such as burns and electric shocks. Injury severity was evaluated with the ISS and GCS as described above. The occurrence of surgical procedures related to the injury was documented. Symptom severity and diagnoses of PTSD were gauged by the Clinician-Administered PTSD Scale (CAPS), applying the Diagnostic and Statistical Manual of Mental Disorders, 5th Edition (DSM-V), criteria (CAPS-5) [22]. Symptom severity for each of the 20 DSM-V PTSD symptoms was determined by multiplying the frequency (Likert Scale, ranging from 0 to 4) by the intensity (Likert Scale, ranging from 0 to 4) of the assessed symptoms, with the total severity score representing the overall severity of PTSD symptoms. This structured clinical interview is among the most widely used tools for evaluating PTSD and has demonstrated high reliability and validity [23]. The presence of a dissociative subtype of PTSD was evaluated within the framework of the CAPS-5 assessment. Higher scores on ISS and CAPS-5 and lower scores on GCS indicate more pronounced symptomatology.

#### 2.2.2. Sociodemographic Characteristics

The following baseline sociodemographic characteristics were collected: age, sex, duration of education, marital status (currently married or not), cohabitation status (living alone or not), religion (religious observance or not), occupational state (current employed or not), and monthly income (above or below 3,000 USD).

#### 2.2.3. Pre-Trauma Characteristics

Several pre-trauma characteristics were assessed to gain insights into participants' backgrounds and potential factors that could contribute to their responses following severe physical injury. Prior histories of psychiatric disorders were documented, including depressive disorders, panic disorder, agoraphobia, social phobia, and generalized anxiety disorder. Participants' experiences of previous lifetime traumatic events were examined using the LEC [21], with the occurrence of at least one type of event categorized as present for analysis purposes. Instances of previous suicidal attempts were identified, defined as intentional self-harm with some intention to die, regardless of objective lethality [24]. A number of physical disorders were assessed using a questionnaire covering 15 systems or diseases. Personality traits were evaluated by the Big Five Inventory [25] and were clustered into resilient and vulnerable types using a standard approach (detailed in the Supplementary Materials Personality Assessments section). Childhood abuse experiences were evaluated with the Nemesis Childhood Trauma Interview [26], which encompassed emotional/psychological, physical, and sexual abuse before the age of 16. A broad definition of “childhood abuse” (having at least one type of abuse) was used for the analysis. Levels of resilience and social support were measured by the Connor–Davidson Resilience Scale (CDRS) [27] and the Multidimensional Scale of Perceived Social Support (MSPSS), respectively. Smoking status and histories were evaluated and divided into current smoking or not. Alcohol-related problems were screened by the Alcohol Use Disorders Identification Test (AUDIT) [28]. Body mass index was calculated. Lower scores on CDRS and higher scores on MSPSS and AUDIT indicate higher symptomatology.

#### 2.2.4. Peri-Trauma Characteristics

During the peri-trauma period (from the index injury to the baseline evaluation), participants' symptoms and functional status were assessed using various evaluation scales. Depressive and anxiety symptoms were evaluated by the Hospital Anxiety Depression Scale-Depression subscale (HADS-D) and Anxiety subscale (HADS-A) [29], respectively; physical function by the Modified Barthel Index (MBI) [30]; subjective cognitive difficulties by the Perceived Deficits Questionnaire-Depression (PDQD) [31]; and hypochondriacal fears by the Illness Attitude Scale (IAS) [32]. Higher scores on HADS-D, HADS-A, PDQD, and IAS and lower scores on MBI indicate more severe symptomatology. Vital signs, including systolic and diastolic blood pressures and heart rate, were measured at the time of baseline evaluation to check physiological status.

### 2.3. Follow-up Evaluations

CAPS-5 was administered to track the progression of symptoms and the presence of PTSD over a 2-year period. The interviews were carried out via telephone, a method that has been demonstrated to be as valid and reliable as face-to-face interviews [33].

### 2.4. Statistical Analysis

Since the delayed onset type of PTSD is defined as PTSD development at least 6 months after the traumatic events, participants evaluated at least once after both baseline and 3-month evaluations comprised the analyzed sample. From a previous longitudinal study on PTSD following traumatic injury [16], we assumed that there would be distinct PTSD trajectory groups that could be derived from CAPS-5 scores across 5 time points over 2 years. Latent class growth analysis (LCGA) was conducted to identify these trajectory groups. We selected LCGA over latent growth mixture modeling or hierarchical linear modeling due to its robust handling of missing data through maximum likelihood estimation. This method efficiently utilizes all available data points without the need for imputation, which proved particularly advantageous given the missing follow-up assessments in our dataset. This approach ensures the integrity of our class assignments and the robustness of our findings. The best-fitting unconditional model was identified by comparing the model fit of progressive numbers of classes (detailed in Supplementary Materials LCGA for PTSD Trajectories section, Figure S1 and Table S1). Baseline data on sociodemographic characteristics, the trauma itself, and pre- and peri-trauma characteristics were compared between the derived trajectory groups using analysis of variance or *χ*^2^ tests. Given that the sample size for each trajectory group was limited, categorical variables were collated into binary variables. Specifically, the four accidental injury types were categorized as traffic-related injuries vs. other injuries, considering the distribution of injury types. Those characteristics significantly associated with trajectory groups (*P*  < 0.05) were entered into logistic regression analyses. These models were used both in total, considering all trajectory groups collectively, and separately for each individual trajectory group to identify and distinguish independent predictors that influence the likelihood of individuals belonging to specific PTSD trajectory groups. Corrections for multiple comparisons were not applied, as the primary goal of these analyses was to broadly identify potential predictors across different PTSD trajectory groups. This approach aims to minimize the risk of overlooking significant variables, thereby reducing the likelihood of Type II errors. All statistical tests were two-sided with a significance level of 0.05. Statistical analyses were carried out using the SPSS 21.0 and STATA 12.0 software.

## 3. Results

### 3.1. Recruitment and Patient Flow

**Figure 1:**
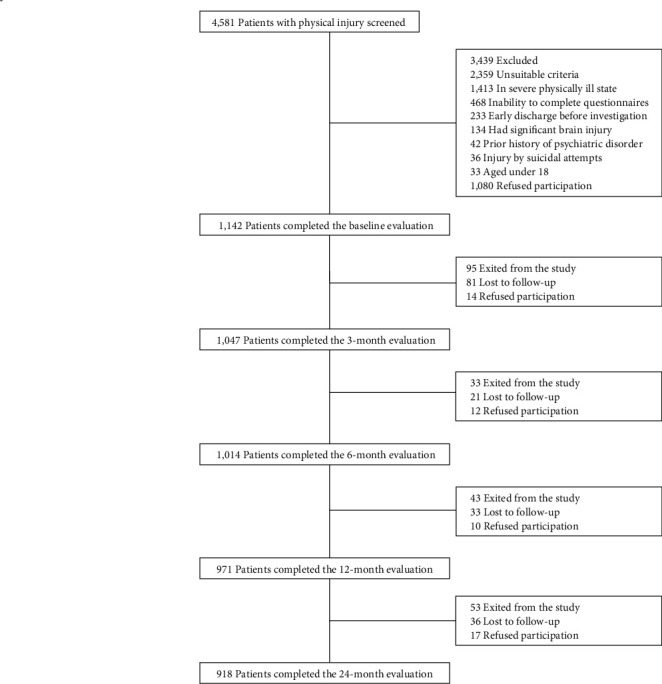
Recruitment and patient flow.

The flow of patients from the baseline assessment to the 24-month follow-up is illustrated in Figure 1. Of 4,581 patients screened, 1,142 met the eligibility criteria and agreed to participate. Of 1,142 patients evaluated at baseline, 1,047 (91.7%) were successfully followed up at the 3-month point, 1,014 (88.8%) at the 6-month point, 971 (85.0%) at the 12-month point, and 918 (80.4%) at the 24-month point. The mean follow-up times were 3.4 months (SD = 0.4), 6.6 months (SD = 0.4), 12.6 months (SD = 0.4), and 24.4 months (SD = 0.6), reflecting both the scheduled assessment intervals and the actual variability in when these assessments were conducted. Overall, 1,014 patients were followed up at least once after the baseline and 3-month evaluations, constituting the analyzed sample. As previously detailed, we employed LCGA, which utilizes maximum likelihood estimation to handle missing data. This statistical approach allows for the utilization of all available data points for each individual, efficiently managing missing values without necessitating imputation. There were no statistical differences in any baseline characteristic between the 1,014 patients included and the 128 who did not follow-up after the 3-month evaluations (all *P*-values > 0.1). The mean (SD) age of participants was 56.8 (17.1) years. Of the participants, 312 (30.8%) were female. The average (SD) educational attainment was 10.7 (4.1) years. Additionally, 66.7% of participants were married, 15.0% lived alone, 41.7% observed a religion, 82.1% were employed, and 42.7% reported a monthly income of at least 3,000 USD.

### 3.2. PTSD Trajectory Groups

**Figure 2:**
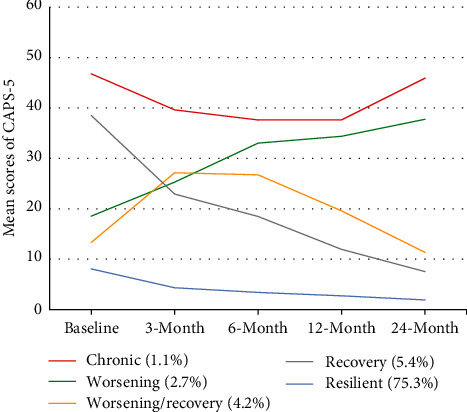
Five trajectories of post-traumatic stress disorder (PTSD) following physical injury. Data are mean scores of the Clinician Administered PTSD Scale for Diagnostic and Statistical Manual of Mental Disorders, 5th edition (CAPS-5).

In the baseline assessment, 75 (7.4%) of 1,014 patients were diagnosed as having PTSD using CAPS-5, and their mean (SD) PTSD severity was 39.5 (10.5) points. After a 2-year follow-up period, 35 (3.8%) of 918 patients diagnosed using CAPS-5 were identified, with a mean (SD) PTSD severity of 40.1 (11.9) points. The LCGA identified a five-cluster model as the best-fitting model characterized by its efficiency, parsimony, and capacity to delineate five distinct PTSD trajectories (detailed in the online supplement). The mean scores of the CAPS-5 for these trajectory groups are displayed in Figure 2. Of note, at all evaluation points, mean CAPS-5 scores were significantly higher (with all *P*-values < 0.001) for each of the four PTSD trajectory groups when compared to the resilient group. The shape of these trajectories closely resembled those observed in a prior study [16]. Consequently, we opted to employ the same group names as follows: (i) resilient group (*N* = 860, 84.8%), exhibiting consistently low levels of PTSD symptoms from baseline to the last follow-up assessment; (ii) worsening/recovery group (*N* = 48, 4.7%): displayed relatively low levels of PTSD symptoms at baseline, which then increased but subsequently decreased by the last follow-up; (iii) worsening group (*N* = 31, 3.1%) with initially relatively low levels of PTSD symptoms at baseline then experiencing an increase in symptoms during the follow-up period; (iv) recovering group (*N* = 62, 6.1%), exhibiting relatively high levels of PTSD symptoms at baseline, which subsequently decreased during the follow-up assessments; and (v) chronic group (*N* = 13; 1.3%), consistently displaying high levels of PTSD symptoms from baseline through to the last follow-up assessment.

### 3.3. Baseline Characteristics by PTSD Trajectory Groups

Table 1 presents a comparison of baseline characteristics among the five distinct PTSD trajectory groups. Significant group differences were observed in age, sex, education, previous psychiatric disorders, previous traumatic events, number of physical disorders, childhood abuse, injury type, dissociative subtype, and scores on HADS-A, HADS-D, and PDQD.

### 3.4. Independent Predictors of PTSD Trajectory Groups

These 12 variables were entered into logistic regression models, the results of which are presented in Table 2. Being in any significant PTSD trajectory group compared to the resilient group was predicted by baseline higher education, previous traumatic events, traffic-related injury type, dissociative PTSD subtype, and higher scores on HADS-A and HADS-D. The analysis was then conducted separately for each specific trajectory group compared to the resilient group, revealing distinct and varying results for each subgroup. The worsening/recovery trajectory was predicted by previous traumatic events and traffic-related injury type; the worsening trajectory by higher education and higher scores on HADS-D; the recovering trajectory by female sex, childhood abuse, traffic-related injury type, dissociative subtype, and higher scores on HADS-A and HADS-D; and chronic trajectory by dissociative subtype and higher scores on HADS-A.

## 4. Discussion

The principal findings of this 2-year prospective longitudinal study involving patients with severe physical injuries are twofold. First, the study confirmed the existence of five distinct trajectories of PTSD symptoms over the 2-year follow-up period. Second, it was found that each of these trajectories was predicted by different baseline characteristics.

Our identification of five trajectory groups and their longitudinal patterns closely align with the results from a previous longitudinal study [16]. Both studies shared a similar research design, in which participants were drawn from individuals who had experienced physical injuries, and the assessment of PTSD was conducted using the CAPS. The primary distinction between the two studies lies in the duration of the follow-up period. In our study, the follow-up period was 2 years, while the previous study extended to 6 years. Despite this difference in follow-up duration, the number of distinct trajectory groups remained consistent at five in both studies. These findings suggest that the longitudinal courses and prognoses of PTSD following severe physical injuries may indeed differ over time, but the fundamental number of trajectory groups remained the same, regardless of the varying follow-up periods. This consistency in the number of trajectory groups emphasizes the robustness of this classification system for understanding the development of PTSD symptoms in this population.

However, our results differ from those of the previous study by Galatzer-Levy et al. [9], which identified three distinct trajectories (nonremitting, slow remitting, and rapid remitting) over a 15-month follow-up. Foa and Tolin [34] employed the PTSD Symptom Scale interviewer version, based on DSM-IV PTSD criteria, whereas we used the CAPS-5 to evaluate PTSD symptoms. Furthermore, the proportion of participants involved in traffic accidents was 85% in their study, compared to 46% in ours. These methodological and demographic differences may contribute to the discrepancies between our findings. Additionally, two other studies identified four trajectories (resilient, delayed-onset, recovery, and nonremitting) [11, 14]. These studies involved fewer participants (400–500) and shorter follow-up periods (6–12 months) compared to our study, which included over 1,000 participants and a 2-year follow-up. Such shorter follow-up periods may capture initial recovery patterns but could miss later stabilizations or deteriorations.

In the full cohort, the presence of any significant PTSD trajectory was predicted by higher education, previous traumatic events, traffic-related injury type, dissociative subtype of PTSD, and higher scores on HADS-A and HADS-D measured at baseline. Many of these characteristics have been previously found to be significantly associated with PTSD when evaluated at specific time points following the traumatic incidents [35, 36, 37]. On the other hand, the association with higher education contrasts with the results of most previous studies, which typically found no significant association between education level and PTSD [38] or else have reported associations with lower education levels and have explained these by more limited coping mechanisms when dealing with traumatic events [39]. Our findings might reflect links between higher education, greater self-awareness, and higher emotional intelligence [40], facilitating the recognition and reporting of PTSD symptoms, leading to a higher likelihood of diagnosis. However, higher education may also contribute to PTSD in several other ways. Individuals with higher education levels might experience alterations in their self-view when faced with trauma, leading to increased psychological distress [41]. Additionally, difficulties in accessing mental health services due to stigma or lack of time can exacerbate symptoms [42]. Financial pressures, coupled with emotional problems, might also play a significant role [43].

Our study also sought to conduct separate analyses for each trajectory group to identify their respective predictors. As described in Table 2, these predictors differed by trajectory group. For example, the previously mentioned association with higher education was significant only with the worsening trajectory group. This might be explained by individuals with higher levels of education initially coping well shortly after physical injury, but over time, becoming increasingly aware of potential emotional disturbances linked to their injuries. These findings underscore the complexity of the relationship between education, psychological factors, and the development of PTSD, and more research is needed to better understand these dynamics. Furthermore, another predictor of the worsening trajectory was higher baseline depressive symptoms. It has been well documented that comorbid depressive symptoms with severe physical disorders have been associated with both poorer long-term psychological and physical prognoses [44, 45]. This supports the importance of recognizing and addressing depressive symptoms when providing care for individuals at acute phases following severe physical injuries, even if they do not exhibit overt PTSD symptoms.

Our study also found a specific association between previous traumatic events and the worsening/recovery trajectory of PTSD. While prior trauma has been identified as a significant predictor of PTSD in certain studies [35], it did not emerge as a significant predictor in other investigations [46]. One potential explanation for these varying results lies in the timing of assessments following physical injuries. Previous studies have typically assessed individuals within a relatively short timeframe after the traumatic event, ranging from 1 to 12 months. Consequently, these studies may not have captured the longer-term trajectory characterized by worsening/recovery, as observed in the present 2-year follow-up study and a previous 6-year follow-up study [16]. In our study, we found that traffic-related injuries, as opposed to other types such as falls and slips, also emerged as an independent predictor of the worsening/recovery trajectory of PTSD. This suggests that there may be specific factors associated with traffic accidents that contribute to this trajectory. One potential explanation could lie in the fact that traffic accidents often involve complex insurance and legal issues. Other research has shown that traffic-related injuries cause treatment delays, chronic pain, and require related treatment during financial or legal proceedings [47, 48]. Dealing with such matters can be emotionally challenging and may contribute to the development or exacerbation of PTSD symptoms. Importantly, this aspect of trauma-related emotional problems, especially in the context of traffic accidents, has been relatively underexplored in previous research. Further studies are therefore warranted to better understand the relationship between the type of injury, including traffic-related incidents, and the development of PTSD symptoms.

The recovery trajectory in our study was predicted by six baseline factors: female sex, previous childhood abuse, traffic-related accident type, dissociative subtype of PTSD, and higher scores on HADS-A and HADS-D. These factors have previously been identified as significantly associated with PTSD in other studies [35, 36, 37]. In our study, we observed that PTSD symptoms remained considerably elevated until 6 months after the injuries in the recovery trajectory group (Figure 2). Most previous PTSD studies following physical injuries typically assessed PTSD within a 6-month timeframe [6, 38, 46]. Given this overlap in assessment periods, it is plausible that we have identified consistent findings between our study and previous research, particularly within the context of the recovery trajectory group. Among the six factors influencing the recovery trajectory, two—female sex and previous childhood abuse—were uniquely associated with this trajectory. It is widely recognized that women and individuals who have experienced childhood abuse are at a heightened risk of developing PTSD [5, 49]. Interestingly, a previous study that identified similar trajectories to ours reported that female sex was associated not only with the recovery trajectory but also with worsening and chronic trajectories [16]. This discrepancy could be attributed to differences in the follow-up period and the number of baseline predictors considered in the studies. Childhood abuse may render individuals more vulnerable to developing PTSD following traumatic injuries, particularly during the early phases of physical injuries, as indicated by our findings of associations exclusively with the recovery trajectory.

Despite the relatively small number, we identified a group with significant and persistent PTSD symptoms spanning a period of 2 years. This chronic trajectory was predicted by the baseline presence of the dissociative subtype of PTSD and higher scores on the HADS-A. The role of peri-trauma dissociation as a strong predictor for subsequent PTSD has been well-established in numerous studies, even though these assessments were typically conducted over relatively short periods (1–6 months after traumatic events) [36, 46, 50]. Our findings extend this understanding, suggesting that dissociative symptoms experienced in the early phase of physical injury can be a potent predictor of PTSD that persists for at least 2 years. Similarly, peri-trauma anxiety has been recognized as a predictor of PTSD, not only in the acute phase [37] but also in the chronic phase (3 years) following physical injuries [51]. Our findings supported these previous studies, emphasizing the enduring impact of peri-trauma anxiety on the development of PTSD. Considering that both dissociative subtypes and anxiety were also identified as predictors of the recovery trajectory, this underscored the importance of carefully managing and treating patients who exhibit these factors. It is also possible that these two symptoms were commonly associated with early PTSD.

Before drawing a conclusion, several issues should be considered. In our analysis, the entropy value observed was lower compared to some prior studies, suggesting a less distinct separation between classes (see Table S1). This phenomenon is particularly noted in contexts where one class predominantly encompasses a large portion of the study population, as was the case in our study, where over 80% of participants were classified within the “resilient” trajectory. Such a distribution can skew the size and distinctiveness of the remaining classes, occasionally resulting in smaller class sizes. Despite the presence of classes containing fewer than 5% of participants, as shown in Figure S1, these classes were retained due to their distinct clinical profiles and the theoretical relevance to our study aims. The literature suggests a minimum class size of 5% for statistical robustness [52]; however, the unique composition of our sample and the theoretical implications of these smaller classes justified their inclusion. This decision underscores the need for a nuanced interpretation of these results, recognizing the potential limits in generalizability and the importance of further validation in more heterogeneous samples. Additionally, in developing our model to predict PTSD trajectories, we initially considered a comprehensive range of variables informed by theoretical relevance and empirical evidence. However, to avoid overfitting and ensure the stability of our model, we deliberately balanced complexity with interpretability and robustness. We opted for a model that integrates the most significant predictors, maintaining manageability and clarity in our findings. We recognize the potential for future research to explore more complex models with additional variables and interactions, especially within larger datasets where such approaches can be more effectively evaluated.

Strengths of the study include the consecutive recruitment of participants at baseline from all eligible patients who had recently experienced physical injuries, as well as the frequent follow-up assessments reducing the risk of bias arising from heterogeneous examination times. Evaluations and data collection were conducted using a structured research protocol and well-recognized and standardized scales, including the CAPS-5 for PTSD. A broad range of potential risk factors for PTSD were comprehensively investigated at baseline. Participants were followed up to 2 years after physical injuries, which allowed a comprehensive understanding of the PTSD trajectories. Long-term follow-up rates were reasonable, and no evidence of selective attrition was found.

Limitations were that participants were recruited exclusively from a single trauma center, which may limit the generalizability, although this approach maximized consistency in evaluation and follow-up. The number of participants who declined to participate was high during the screening procedure, which could introduce selection bias, potentially affecting the representativeness of our sample. Future research could aim to identify and mitigate barriers to participation to ensure a more representative sample of trauma survivors. The study focused on patients who were hospitalized for moderate to severe physical injuries, and findings cannot necessarily be generalized to individuals with more minor physical injuries. Subsequent studies should consider including a broader range of injury severities to cover a wider spectrum of trauma experiences and outcomes. The distribution of patients across the trajectory groups was uneven, with relatively few participants, particularly in the chronic trajectory group, which might attenuate statistical power and reduce generalizability. However, a similar pattern was observed in a previous study with a comparable design [16]. The lack of interrater reliability data for the CAPS could impact the consistency of the PTSD assessments. Follow-up evaluations were conducted via telephone interview, although this method has proved to be as valid as face-to-face interviews [33].

In conclusion, five distinctive PTSD trajectories were observed over 2 years following severe physical injuries, and these were predicted by different baseline characteristics. These findings highlighted the heterogeneity of PTSD symptom development and emphasized the importance of considering individual characteristics when assessing and addressing PTSD in patients with severe physical injuries. Furthermore, these observations may help explain the inconsistencies observed in previous studies that have examined predictive factors for PTSD from specific time-point evaluations and may provide valuable insights that can inform the development of tailored preventive and management strategies for this complex issue. Specifically, implementing routine screening for PTSD symptoms in individuals presenting with identified risk factors at healthcare centers can lead to early identification and treatment, potentially altering the trajectory before it worsens. Developing personalized treatment plans based on an individual's specific risk profile and trajectory is crucial, ensuring that interventions are responsive to changes in their condition over time. For those identified at risk due to factors like previous traumatic events or childhood abuse, integrating preventive interventions such as resilience training or stress inoculation training into routine care could help mitigate the development of severe PTSD. Future studies are needed to improve generalizability through multi-center evaluations and include individuals with traumatic events beyond physical injuries.

## Figures and Tables

**Table 1:** Baseline characteristics by post-traumatic stress disorder trajectory for two years in 1,014 patients with physical injuries.

	Resilient (*N* = 860)	Worsening/ recovery (*N* = 48)	Worsening (*N* = 31)	Recovery (*N* = 62)	Chronic (*N* = 13)	Statistical coefficients	*P*-value^a^
Sociodemographic characteristics							
Age, mean (SD) years	57.3 (17.4)	54.9 (13.8)	48.8 (13.4)	56.7 (16.7)	47.0 (14.4)	*F* = 3.156	**0.014**
Sex, *N* (%) female	247 (28.7)	14 (29.2)	11 (35.5)	34 (54.8)	6 (46.2)	*χ* ^2^ = 20.382	**<0.001**
Education, mean (SD) years	10.6 (4.1)	11.1 (3.0)	13.1 (3.6)	10.7 (4.4)	12.6 (2.3)	*F* = 3.697	**0.005**
Marital status, *N* (%) unmarried	292 (34.0)	13 (27.1)	8 (25.8)	19 (30.6)	6 (46.2)	*χ* ^2^ = 2.946	0.567
Living alone, *N* (%)	136 (15.8)	4 (8.3)	3 (9.7)	9 (14.5)	0 (0.0)	*χ* ^2^ = 5.117	0.275
Religious observance, *N* (%)	344 (40.0)	24 (50.0)	16 (51.6)	30 (48.4)	9 (69.2)	*χ* ^2^ = 8.828	0.066
Unemployed status, *N* (%)	156 (18.1)	6 (12.5)	4 (12.9)	14 (22.6)	1 (7.7)	*χ* ^2^ = 3.364	0.499
Monthly income, *N* (%) < 3,000 USD	502 (58.4)	20 (41.7)	17 (54.8)	36 (58.1)	6 (46.2)	*χ* ^2^ = 5.950	0.203
Pre-trauma characteristics							
Previous psychiatric disorders, *N* (%)	53 (6.2)	7 (14.6)	3 (9.7)	8 (12.9)	2 (15.4)	*χ* ^2^ = 9.908	**0.042**
Previous traumatic events, *N* (%)	30 (3.5)	5 (10.4)	3 (9.7)	4 (6.5)	4 (30.8)	*χ* ^2^ = 29.008	**<0.001**
Previous suicidal attempt, *N* (%)	16 (1.9)	2 (4.2)	0 (0.0)	2 (3.2)	0 (0.0)	*χ* ^2^ = 2.640	0.620
Number of physical disorders, mean (SD)	2.0 (2.1)	1.9 (2.0)	0.9 (1.2)	1.7 (1.9)	1.2 (1.4)	*F* = 2.575	**0.036**
Vulnerable personality, *N* (%)	373 (43.4)	22 (45.8)	14 (45.2)	26 (41.9)	8 (61.5)	*χ* ^2^ = 1.913	0.752
Any childhood abuse, *N* (%)	47 (5.5)	3 (6.3)	2 (6.5)	7 (11.3)	2 (15.4)	*χ* ^2^ = 5.545	**0.027**
Connor–Davidson Resilience Scale, mean (SD) scores	65.4 (15.5)	66.6 (17.1)	67.7 (16.6)	62.0 (17.6)	59.7 (15.5)	*F* = 1.331	0.256
Multidimensional scale of perceived social support, mean (SD) scores	34.7 (9.9)	34.8 (10.4)	38.4 (10.7)	35.2 (9.9)	36.4 (11.8)	*F* = 1.083	0.364
Current smoker, *N* (%)	233 (27.1)	19 (39.6)	9 (29.0)	13 (21.0)	5 (38.5)	*χ* ^2^ = 5.731	0.220
Alcohol use disorders identification test, mean (SD) scores	10.4 (10.0)	10.5 (10.5)	11.1 (9.9)	7.7 (9.7)	6.2 (7.1)	*F* = 1.607	0.170
Body mass index, mean (SD)	23.6 (3.4)	24.3 (3.1)	24.1 (3.4)	23.3 (3.5)	22.6 (3.0)	*F* = 1.006	0.403
Trauma-related characteristics							
Injury type, *N* (%) traffic-related	374 (43.5)	30 (62.5)	14 (45.2)	37 (59.7)	6 (46.2)	*χ* ^2^ = 12.385	**0.015**
Injury Severity Score, mean (SD) scores	14.5 (5.7)	14.6 (5.6)	16.9 (6.5)	15.3 (6.4)	15.1 (7.3)	*F* = 1.649	0.160
Glasgow Coma Scale, mean (SD) scores	14.8 (0.7)	14.9 (0.6)	14.8 (0.6)	14.8 (0.7)	14.9 (0.4)	*F* = 0.045	0.996
Got surgery for the injury, *N* (%)	440 (51.2)	30 (62.5)	21 (67.7)	37 (59.5)	9 (69.2)	*χ* ^2^ = 8.092	0.088
Dissociative subtype, *N* (%)	85 (9.9)	8 (16.7)	5 (16.1)	37 (59.7)	8 (61.5)	*χ* ^2^ = 143.457	**<0.001**
Peri-trauma assessment scales and measurements, mean (SD)							
Hospital Anxiety Depression Scale-anxiety subscale scores	2.5 (2.9)	3.7 (3.7)	4.2 (3.3)	8.9 (4.5)	11.5 (4.9)	*F* = 90.393	**<0.001**
Hospital Anxiety Depression Scale-depression subscale scores	4.8 (4.5)	7.0 (4.8)	6.9 (4.8)	12.5 (4.5)	11.4 (5.0)	*F* = 50.474	**<0.001**
Mini-Mental State Examination scores	24.4 (5.3)	24.1 (4.5)	25.7 (3.8)	24.1 (4.8)	24.5 (7.7)	*F* = 0.555	0.696
Modified Barthel Index scores	58.7 (31.3)	51.1 (33.0)	56.2 (35.2)	49.4 (30.9)	59.9 (35.0)	*F* = 1.838	0.119
Perceived Deficits Questionnaire-Depression scores	6.2 (11.3)	8.5 (11.1)	5.6 (11.7)	11.9 (12.5)	12.8 (18.3)	*F* = 4.971	**0.001**
Illness Attitude Scale scores	59.3 (20.4)	65.3 (22.0)	53.8 (15.7)	63.6 (22.1)	63.3 (23.5)	*F* = 2.281	0.059
Systolic blood pressure (mmHg)	119.8 (13.7)	111.6 (14.5)	119.4 (10.8)	118.3 (12.6)	114.8 (10.6)	*F* = 1.125	0.343
Diastolic blood pressure (mmHg)	72.3 (9.2)	68.7 (9.4)	73.3 (8.6)	70.6 (8.8)	69.9 (7.0)	*F* = 1.633	0.164
Heart rate per minute	78.3 (11.2)	77.9 (11.1)	77.2 (11.5)	78.9 (10.2)	80.8 (9.9)	*F* = 0.307	0.874

^a^Analysis of variance or *χ*^2^ tests, as appropriate. Bold style indicates statistical significance.

**Table 2:** Baseline predictors of post-traumatic stress disorder trajectory for 2 years in 1,014 patients with physical injuries.

	Any^a^ (*N* = 154)	Worsening/recovery (*N* = 48)	Worsening (*N* = 31)	Recovery (*N* = 62)	Chronic (*N* = 13)
Higher age	0.99 (0.98–1.01)	0.99 (0.97–1.02)	0.99 (0.96–1.02)	0.99 (0.97–1.03)	0.97 (0.91–1.04)
Female sex	1.26 (0.84–1.90)	0.83 (0.41–1.68)	1.41 (0.61–3.23)	**2.25 (1.09–4.65)** *⁣* ^*∗*^	2.25 (0.42–11.91)
Higher education	**1.09 (1.02–1.15)** ^**†**^	1.05 (0.96–1.16)	**1.20 (1.05–1.38)** ^**†**^	1.08 (0.98–1.20)	1.31 (0.97–1.78)
Had previous psychiatric disorders	0.78 (0.41–1.48)	1.67 (0.65–4.29)	0.58 (0.15–2.26)	0.39 (0.12–1.22)	0.14 (0.01–2.42)
Had previous traumatic events	**2.46 (1.11–5.46)** *⁣* ^*∗*^	**2.76 (1.00–7.86)** *⁣* ^*∗*^	3.05 (0.79–11.77)	0.57 (0.09–3.55)	6.43 (0.54–76.39)
Higher number of physical disorders	0.92 (0.82–1.03)	1.01 (0.85–1.21)	0.76 (0.57–1.02)	0.92 (0.74–1.13)	0.82 (0.46–1.48)
Had any childhood abuse	1.35 (0.65–2.80)	0.94 (0.26–3.42)	0.97 (0.21–4.60)	**5.15 (1.61–16.44)** ^**†**^	1.68 (0.08–34.54)
Traffic-related injury type	**1.48 (1.00–2.19)** *⁣* ^*∗*^	**2.23 (1.19–4.19)** *⁣* ^*∗*^	0.81 (0.37–1.79)	**2.91 (1.40–6.07)** ^**†**^	1.45 (0.26–8.25)
Present dissociative subtype	**2.28 (1.44–3.59)** ^**‡**^	1.32 (0.57–3.06)	1.10 (0.38–3.22)	**6.77 (3.34–13.74)** ^**‡**^	**5.42 (1.15–25.43)** *⁣* ^*∗*^
Higher Hospital Anxiety Depression Scale-anxiety subscale scores	**1.20 (1.12–1.278)** ^**‡**^	1.05 (0.94–1.18)	1.11 (0.96–1.29)	**1.33 (1.19–1.48)** ^**‡**^	**1.60 (1.25–2.04)** ^**‡**^
Higher Hospital Anxiety Depression Scale-depression subscale scores	**1.101 (1.04–1.16)** ^**†**^	1.07 (0.98–1.17)	**1.12 (1.00–1.26)** *⁣* ^*∗*^	**1.16 (1.05–1.27)** ^**†**^	1.04 (0.85–1.27)
Higher Perceived Deficits Questionnaire-depression scores	1.00 (0.99–1.02)	1.01 (0.98–1.04)	1.00 (0.96–1.04)	1.01 (0.98–1.04)	1.04 (0.98–1.10)

^a^Any refers to all trajectory groups. *⁣*^*∗*^*P* < 0.05; ^**†**^*P* < 0.01; ^**‡**^*P* < 0.001. Bold style indicates statistical significance. Data are odds ratios (confidence intervals) compared with the resilient group (*N* = 860).

## Data Availability

The data that support the findings of the study are available from the corresponding author (Jae-Min Kim) upon reasonable request.

## Supplementary Materials

Supplementary Materials The supplementary material includes information on Personality assessments and Latent Class Growth Analysis (LCGA) for post-traumatic stress disorder (PTSD) trajectories.
