# Supplementary material for: Predictors of 2-Year Trajectory of Post-Traumatic Stress Disorder Following Physical Injury
Source: Depress Anxiety. 2024 Sep 2;2024:5570405. doi: 10.1155/2024/5570405 (PMC11919229; doi:10.1155/2024/5570405)
Supplement: Supplementary Materials — The supplementary material includes information on Personality assessments and Latent Class Growth Analysis (LCGA) for post-traumatic stress disorder (PTSD) trajectories. [file 5570405.f1.docx]

Supplementary materials

**Personality assessments**

**Latent class growth analysis (LCGA) for post-traumatic stress disorder (PTSD) trajectories**

**Table S1.** Goodness of fit criteria for 3~7 class models of post-traumatic stress disorder trajectories.

**Figure S1.** Plots of unselected classes of post-traumatic stress disorder (PTSD) trajectories. Data are mean scores of the Clinician Administered PTSD Scale for Diagnostic and Statistical Manual of mental disorders, 5th edition (CAPS-5).

**References**

**Personality assessments**

- *This is the detailed descriptions on the way of Personality assessments in the Methods section.*

Personality was assessed by the Big Five Inventory (John and Srivastata, 1999). Using the typological approach, personality cluster was identified using cluster analysis. Result of identical analyses with the two-step procedure specified by Asendorpf et al. (2001), two personality types were identified: resilient and vulnerable. Ward's hierarchical clustering procedure was applied for the initial solution; then, iterative k-means clustering, using Ward's method to define the initial cluster centres, was performed. Each case was assigned to a cluster based on the Euclidean distance from the cluster means; all data was z-standardised to determine the Euclidean distance. The largest shifts in cluster coefficients were observed in the transition from the two- to one-cluster solution; therefore, a two-cluster solution was accepted as the best initial solution. The cluster centres derived from the initial solution were used to implement non-hierarchical k-means clustering.

To evaluate the replicability of the final solutions, the method of Asendorpf et al. (2001) was used. Briefly, all cases were randomly split into halves and the full two-step procedure was applied to each half. Next, the participants from each half were assigned to new clusters according to the cluster centres of the other half of the sample. These new clusters were then compared to determine if they agreed with the original clusters according to Cohen's k. A kappa value > 0.60 was required as evidence of replication; the present results satisfied this criterion (replicability coefficients with Cohen's k: 0.833). Compared with the second cluster, the first cluster was characterized by significantly higher extraversion, higher agreeableness, higher conscientiousness, but lower neuroticism (all *p* < 0.001). As defined and labeled in previous studies, the first cluster was labeled as resilient type and the second was vulnerable type (Wardenaar et al., 2014, De Fruyt, 2002).

**Latent class growth analysis (LCGA) for post-traumatic stress disorder (PTSD) trajectories**

- *This is the detailed descriptions on the process and results of LCGA for PTSD trajectories in the Statistical analysis section.*

LCGA was used to model PTSD trajectories over time based on scores from the Clinician Administered PTSD Scale for Diagnostic and Statistical Manual of Mental Disorders, 5th edition (CAPS-5) at five time points (baseline, month 3, 6, 12, 24). The best-fitting trajectory model was determined by comparing models with varying numbers of classes and components (linear only vs. linear and quadratic) using established model fit criteria, including Akaike's information criterion (AIC), Bayesian information criterion (BIC), Bootstrap-Loglikelihood Ratio Test (BLRT), and entropy values. Additionally, considerations of parsimony, theoretical relevance, and clinical significance were taken into account in selecting the optimal model (Wang & Wang, 2012).

The results of the model fit for PTSD trajectories are summarized in Table S1. Models that included both linear and quadratic components were found to provide a better fit compared to models with linear components only. Similar to a previous study (Bryant et al., 2015), the 5-class solution demonstrated the best fit, as evidenced by substantial decreases in AIC and BIC from the 3-class to the 5-class model, with only marginal improvement from the 5-class to the 7-class model. The BLRT consistently indicated significant improvement with the addition of new classes, but the 6-class model did not yield a substantively distinct class. The entropy value for the 5-class model was intermediate, falling between values for models with fewer and more classes. Taking all these findings into consideration, the 5-class solution was chosen as the most appropriate model.

| **Table S1.** Goodness of fit criteria for 3~7 class models of post-traumatic stress disorder trajectories. | | | | |
| --- | --- | --- | --- | --- |
|  | AIC | BIC | BLRT  (p-value) | Entropy |
| Linear |  |  |  |  |
| 3 class | 15332.9 | 15441.3 | <0.001 | 0.756 |
| 4 class | 15243.3 | 15340.2 | <0.001 | 0.762 |
| 5 class | 15144.3 | 15247.0 | <0.001 | 0.765 |
| 6 class | 15101.1 | 15198.4 | 0.063 | 0.747 |
| 7 class | 15087.6 | 15164.7 | 0.090 | 0.738 |
| Linear + quadratic |  |  |  |  |
| 3 class | 15212.5 | 15317.0 | <0.001 | 0.777 |
| 4 class | 15109.1 | 15182.8 | <0.001 | 0.780 |
| 5 class | 15006.4 | 15107.3 | <0.001 | 0.783 |
| 6 class | 15093.3 | 15189.7 | 0.018 | 0.774 |
| 7 class | 15080.7 | 15156.0 | 0.045 | 0.765 |
| AIC: Akaike’s information criterion; BIC: Bayesian information criterion; BLRT: Bootstrap-Loglikelihood Ratio Test (BLRT) | | | | |

**Figure S1.** Plots of unselected classes of post-traumatic stress disorder (PTSD) trajectories. Data are mean scores of the Clinician Administered PTSD Scale for Diagnostic and Statistical Manual of mental disorders, 5^th^ edition (CAPS-5).


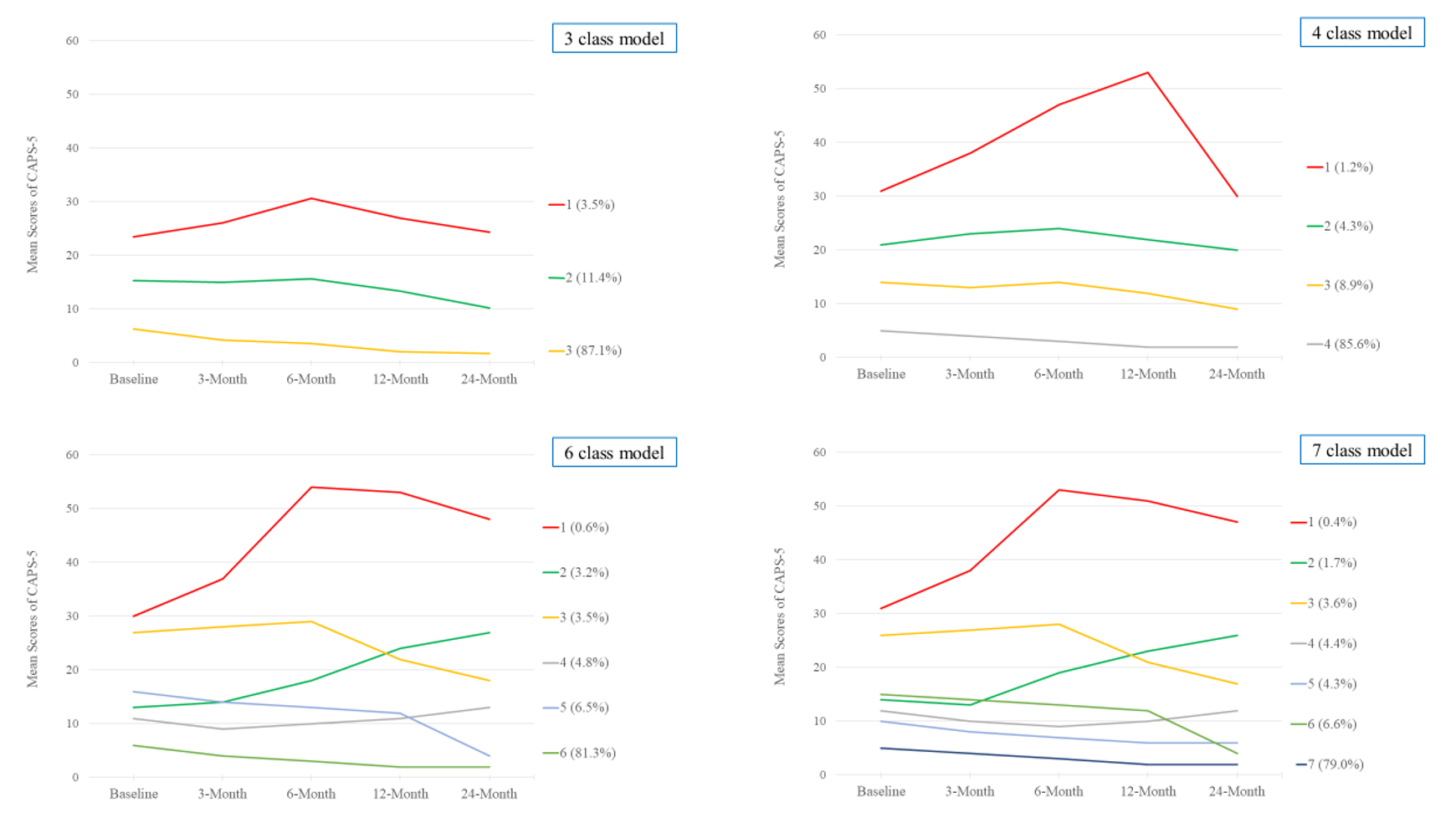


**References**

- *This is the detailed references of the Supplementary materials.*

Asendorpf, J.B., Borkenau, P., Ostendorf, F., van Aken, M.A., 2001. Carving personality description at its joints: Confirmation of three replicable personality prototypes for both children and adults. Eur. J. Pers. 15, 169-198.

Bryant RA, Nickerson A, Creamer M, O'Donnell M, Forbes D, Galatzer-Levy I, McFarlane AC, Silove D. Trajectory of post-traumatic stress following traumatic injury: 6-year follow-up. Br J Psychiatry. 2015 May;206(5):417-23. doi: 10.1192/bjp.bp.114.145516. Epub 2015 Feb 5. PMID: 25657356.

De Fruyt, F., 2002. A Person-Centered Approach to P–E Fit Questions Using a Multiple-Trait Model. J. Vocat. Behav. 60, 73–90.

John, O.P., Srivastata, S, 1999. The Big Five Trait taxonomy: history, measurement, and theoretical perspertives, in: Pervin, L.A., John, O.P (Eds), Handbook of Personality. 2nd ed. The Guilford Press, New York, pp. 102-138.

Wang, J., & Wang, X, 2012. Structural equation modeling: Applications using mplus. New York, NY: John Wiley.

Wardenaar, K.J., Conradi, H.J., Bos, E.H., de Jonge, P., 2014. Personality modulates the efficacy of treatment in patients with major depressive disorder. J. Clin. Psychiatry. 75, e916-923.
